# Cuticular hydrocarbon reception by sensory neurons in basiconic sensilla of the Japanese carpenter ant

**DOI:** 10.3389/fncel.2023.1084803

**Published:** 2023-02-06

**Authors:** Hidehiro Watanabe, Shoji Ogata, Nonoka Nodomi, Kosuke Tateishi, Hiroshi Nishino, Ryosuke Matsubara, Mamiko Ozaki, Fumio Yokohari

**Affiliations:** ^1^Department of Earth System Science, Fukuoka University, Fukuoka, Japan; ^2^Research Institute for Electronic Science, Hokkaido University, Sapporo, Japan; ^3^Department of Chemistry, Graduate School of Science, Kobe University, Kobe, Japan; ^4^Department of Biology, Graduate School of Science, Kobe University, Kobe, Japan; ^5^KYOUSEI Science Center for Life and Nature, Nara Women’s University, Nara, Japan

**Keywords:** ant, cuticular hydrocarbons, basiconic sensilla, sensory neurons, nestmate recognition, social insect

## Abstract

To maintain the eusociality of a colony, ants recognize subtle differences in colony-specific sets of cuticular hydrocarbons (CHCs). The CHCs are received by female-specific antennal basiconic sensilla and processed in specific brain regions. However, it is controversial whether a peripheral or central neural mechanism is mainly responsible for discrimination of CHC blends. In the Japanese carpenter ant, *Camponotus japonicus*, about 140 sensory neurons (SNs) are co-housed in a single basiconic sensillum and receive colony-specific blends of 18 CHCs. The complexity of this CHC sensory process makes the neural basis of peripheral nestmate recognition difficult to understand. Here, we electrophysiologically recorded responses of single basiconic sensilla to each of 18 synthesized CHCs, and identified CHC responses of each SN co-housed in a single sensillum. Each CHC activated different sets of SNs and each SN was broadly tuned to CHCs. Multiple SNs in a given sensillum fired in synchrony, and the synchronicity of spikes was impaired by treatment with a gap junction inhibitor. These results indicated that SNs in single basiconic sensilla were electrically coupled. Quantitative analysis indicated that the Japanese carpenter ants have the potential to discriminate chemical structures of CHCs based on the combinational patterns of activated SNs. SNs of ants from different colonies exhibited different CHC response spectra. In addition, ants collected from the same colony but bred in separate groups also exhibited different CHC response spectra. These results support the hypothesis that the peripheral sensory mechanism is important for discrimination between nestmate and non-nestmate ants.

## Introduction

In eusocial insects, chemical communication via pheromones plays important roles in regulating the sophisticated organization of their societies (Wyatt, 2014). One group of pheromones that is extremely important in social insects is long-chained and low-volatility hydrocarbons found on the cuticular surface, collectively known as cuticular hydrocarbons (CHCs) (Blomquist and Bagnères, 2010). The composition of CHCs in ants differs not only between members of different colonies, but also between castes within the same colony (Blomquist and Bagnères, 2010). Based on subtle differences in CHC blends, an ant can quickly and reliably discriminate colony membership via antennal contact, and then exhibit familiar or aggressive behaviors to a nestmate or non-nestmate ant, respectively (Hölldobler and Wilson, 1990). Despite the importance of CHCs for colony organization, the neural mechanisms of how an ant receives and processes CHC blends are still controversial.

On ant antennae, eight morphological types of sensilla are abundantly distributed (Nakanishi et al., 2009; Ramirez-Esquivel et al., 2014). Among them, CHCs are received by sensory neurons (SNs) in the female-specific antennal basiconic sensilla (Ozaki et al., 2005; Sharma et al., 2015; Ghaninia et al., 2017). SNs in basiconic sensilla selectively project to the female-specific cluster of glomeruli (T6 glomeruli) of the first-order olfactory center (the antennal lobe) in the brain (Zube and Rossler, 2008; Zube et al., 2008; Nakanishi et al., 2009, 2010; McKenzie et al., 2016; Trible et al., 2017). The basiconic sensillum exhibits olfactory pores on its cuticular surface (Nakanishi et al., 2009; Takeichi et al., 2018; Gellert et al., 2022), and SNs in basiconic sensilla expressing olfactory receptors (Trible et al., 2017). Therefore, SNs in basiconic sensilla may process CHCs as olfactory cues. However, in the ant brain, the projection neurons from T6 glomeruli terminate in specific regions of the higher brain centers, the mushroom body and lateral horn (Nishikawa et al., 2012), suggesting CHC information is thought to be processed in specific neural circuits independent from other olfactory information.

Currently, two hypotheses of CHC processing for nestmate recognition have been proposed, the “pre-filter hypothesis” and the “template matching hypothesis” (Ozaki and Hefetz, 2014). In the Japanese carpenter ant *Camponotus japonicus*, SNs in basiconic sensilla exhibit strong responses to non-nestmate CHC blends but not to nestmate CHC blends (Ozaki et al., 2005; Kidokoro-Kobayashi et al., 2012). In the pre-filter hypothesis, after chronic exposure of nestmate CHCs, SNs adapt to nestmate CHC blends (Ozaki et al., 2005; Mizutani et al., 2021). Alternatively, in *Camponotus floridanus*, SNs in single basiconic sensilla exhibit excitatory responses to both nestmate and non-nestmate CHC blends with different activity patterns and without pronounced response adaptation (Sharma et al., 2015). Therefore, in the template matching hypothesis, an ant might learn the nestmate CHC blend and create a neural template in the brain (Neupert et al., 2018). The CHC blend of an encountered ant would be deciphered on the basis of the combination of SN activity patterns and compared with a neural template in the higher brain centers. There is a critical difference between the two hypotheses, especially in the peripheral sensory processing of CHCs. Both hypotheses have been proposed based on electrophysiology data of mass responses of multiple SNs in single basiconic sensilla to the nestmate or non-nestmate CHC blend using different ant species (Ozaki et al., 2005; Sharma et al., 2015), and the complexity of this CHC sensory process makes the neural basis of peripheral nestmate recognition difficult to understand. Therefore, to determine the CHC processing in ants, it needs to reveal receptive properties of each SN in single basiconic sensilla to each CHC composing CHC blends for nestmate recognition.

To study CHC processing in ants, we focused on basiconic sensilla of *C. japonicus* as a model. In *C. japonicus*, gas chromatography and mass spectrometry analysis, and behavioral analysis have been identified colony-specific blends of 18 CHCs that are critical for nestmate recognition (Ozaki et al., 2005), and all 18 CHCs have been synthesized (Uebi et al., 2022). Furthermore, morphological features of SNs in single basiconic sensillum have been characterized in *C. japonicus*; approximately 140 SNs whose dendrites connected each other via gap junctions are co-housed in single basiconic sensillum (Nakanishi et al., 2009; Takeichi et al., 2018). In *C. japonicus*, electrophysiological studies have been revealed that multiple SNs in single basiconic sensillum exhibit strong excitatory responses to the non-nestmate CHC blend directly presented to the sensillar tip (Ozaki et al., 2005; Kidokoro-Kobayashi et al., 2012). In this study, we characterized response properties of single SNs in basiconic sensilla to each CHC that *C. japonicus* uses for nestmate recognition. Taken together our new results with previous results obtained by *C. japonicus*, it must help to understand the neural mechanisms underlying nestmate recognition in the ant.

## Materials and methods

### Ants

We used Japanese carpenter ants *C. japonicus*, collected from September to December in 2016 and 2017 within the campus of Fukuoka University, Fukuoka, Japan. We collected worker ants from three colonies, termed nest-A (NA), -B (NB), and -C (NC), which were located within 200 m but separated from each other by a building. In *C. japonicus*, ants from the different colonies exhibit aggressive behavior toward each other but not toward those in the same colony (Ozaki et al., 2005; Mizutani et al., 2021). Ten to twenty ants collected from each of the three colonies were reared together in a plastic chamber at 25°C with a 12-h:12-h light/dark photoperiod. The ants were fed by 500 mM sucrose solution *ad libitum*. After all ants had been used in experiments, more ants were collected. Therefore, we termed recorded basiconic sensilla (BS) based on “colony name (NA, NB, or NC)–sampling time (year. month)–sensilla number (BS1-6).” The sensilla were numbered according to the recording date order.

### Scanning electron microscopy and retrograde staining of SNs

For scanning electron microscopy (SEM), isolated antennae were fixed in 50% acetone and ultrasonically cleaned. The antennae were then post-fixed in 4% OsO_4_ in 50% acetone for 2 h, dehydrated in a graded acetone series, air-dried, and coated with platinum-palladium using an ion sputter (E-1030; Hitachi, Tokyo, Japan). Observations were performed with a field emission scanning electron microscope (S-4800; Hitachi, Tokyo, Japan). Basiconic sensilla were identified based on previous descriptions of *C. japonicus* (Nakanishi et al., 2009).

To visualize SNs in single basiconic sensilla, we performed retrograde staining of antennal afferents using a previously reported method (Nakanishi et al., 2009, 2010). Briefly, the decapitated ant head was fixed on a plate, and the proximal part of an antenna was embedded in low-melting point wax. A borosilicate microelectrode with a fractured tip was filled with a 10% aqueous solution of micro-ruby (dextran tetramethylrhodamine with biotin, 3,000 MW, D-7156, Thermo Fisher Scientific, MA, USA). The antennal nerves were exposed by removing the cuticle surface of the scape and then cut in the normal ant saline (4.8 mM TES, pH 7.4, containing 127 mM NaCl, 6.7 mM KCl, 2 mM CaCl_2_, and 3.5 mM sucrose). The distal cut nerve ends were inserted into glass electrodes so as to make contacts with the dye. After incubation in a dark and moist chamber at 4°C for 48 h, the flagellum of the antenna was cut off and manually sliced with a piece of razorblade. The sliced antenna was fixed in 4% formaldehyde solution at 4°C for 1 day, and then dehydrated in an ascending ethanol series and cleared in methyl salicylate. The cleared specimens were examined with a confocal laser scanning microscope (LSM-510; Carl Zeiss, Jena, Germany). Optical sections of 1,024 × 1,024 pixels captured at intervals of 0.5–1.0 μm thickness at were processed using image processing software (Amira 6.0, Thermo Fisher Scientific, MA, USA).

### Behavioral experiments

To confirm the experimental condition for the CHC stimulation, we examined behavioral responses of tethered ants to vaporized nestmate and non-nestmate CHC extracts (Supplementary Figure 1). Ten to twenty ants sampled from two different colonies were reared in plastic chambers. Before behavioral experiments, the crude extract of CHC extract of each group was obtained by immersing five ants into 2 mL hexane for 1 min (Ozaki et al., 2005). Each remaining ant was tethered using a handmade immobilizing device consisting of a plastic Petri dish with a V-shaped opening in its rim (Lucas et al., 2005). The tethered ants were left in the experimental room for 2–3 h to familiarize the experimental condition.

Just before behavioral experiment, 20 μL of nestmate or non-nestmate CHC extract was soaked with a filter paper (0.5 × 1 cm). After drying hexane, the filter paper was loaded the glass nozzle attached to heated air delivery system (see following sections). After attaching a tethered ant to the experimental apparatus, the ant received heated clean air for 1 min, and then received the heated air through pass the nestmate or non-nestmate CHC extract. We recorded behavioral responses of the tethered ants to a given CHC extract for 1.5 min using a high-speed camera with 100 fps (HAS-220; Ditect, Tokyo, Japan). We determined levels of aggressiveness of the tethered ants according to previous studies as follows; 0: no responses, 1: intense antennal scanning during the stimulus period, 2; mandibles slightly opened, 3; mandibles widely opened, 4; body jerking with open mandibles, 5; gaster twisted forward to spray formic acid (Hölldobler and Wilson, 1990; Lucas et al., 2005; Brandstaetter et al., 2008). When the ant exhibited 3, 4, or 5 behavioral responses during the recording period, we regarded that the ant exhibited the aggressive behavior to the vaporized CHC extract. In experiment 1, eleven tethered ants were stimulated by the non-nestmate CHC extract heated by air with four different temperatures. We evaluated the temperature dependent behavioral changes of individual ants by the Wilcoxon signed-rank test. In experiment 2, 60 tethered ants were received both nestmate and non-nestmate CHC extracts heated by 50°C air. In the experiments, aggressive behaviors induced by two different CHC extracts were statistically compared using the chi-square test. In both experiments, CHCs were presented with interstimulus interval of >5 min.

### Single sensillum recording

For extracellular recordings from single basiconic sensilla, an ice-anesthetized ant was fixed ventral-side-up to a handmade acrylic plate that had been covered with a thin layer of low-melting point wax. To prevent movement, the body and legs were mounted in the wax on the acrylic plates. The antennae were gently fixed using the wax on the plate. An antenna was viewed through a light microscope (BX51WI; Olympus, Tokyo, Japan) at 200 × and 500 × magnification using objectives, LM PlanFI 20 × /0.40 and LM PlanFI 50 × /0.50, respectively.

We performed single sensillum recording (SSR) from an arbitrarily selected basiconic sensillum on the ventral surface of the antenna. For the recording, a silver wire indifferent electrode (0.2 mm in diameter) was manually inserted into the head capsule around the ipsilateral compound eye, and an electrolytically sharpened tungsten electrode was inserted into the base of the selected sensillum using a micromanipulator. After observing spontaneous spike activity, CHCs were presented to recorded antenna. Electrical events were processed by a pre-amplifier (MEZ-8201; Nihon Kohden, Tokyo, Japan) and a main AC/DC amplifier (EX1; Dagan Corporation, Minneapolis, MN, USA). Spikes presented as DC potentials were digitized and recorded using a Power Lab data acquisition system at a sampling rate of 20 kHz (Power Lab 8/35; AD Instruments Japan Inc., Nagoya, Japan). It is technically difficult to complete a long-time recording from single sensilla in ants. In the present study, we performed SSRs from totally about a hundred of ants, and recorded more than 50 basiconic sensilla. However, half of them were useless, because they allowed us to get only a couple of CHC responses. Finally, we analyzed CHC responses of 23 sensilla in which we successfully recorded during the long-time period with analyzable signal to noise ratio. These 23 sensilla were obtained from different ants belonging to one of the five different ant groups.

### CHCs and their delivery system

As stimulants for the SSR, we chose a panel of 18 CHCs that *C. japonicus* uses for nestmate recognition and one general odor octanal, C8al (Table 1). Our preliminary experiments revealed that C8al is one of the most effective odorants for activating multiple SNs in basiconic sensilla (data not shown). Among the 18 CHCs, seven were commercially available, but 11 needed to be synthesized (Table 1). Details of CHC synthesis have been reported (Uebi et al., 2022). All CHCs and C8al were diluted to 1 mM in paraffin oil, and the concentration of CHC elicited sufficient excitatory responses in SNs of basiconic sensilla (Supplementary Figure 2). To deliver a CHC to an antenna, 1 mL of a given CHC solution was placed in a glass tube (1 cm in diameter) and was heated in a heating block at 80°C (HF100; Yamato, Tokyo, Japan) to vaporize the CHCs, which were then delivered as odor stimuli to an antenna positioned approximately 1 cm apart from the tip of the tube. CHCs were randomly presented to an antenna, and each CHC was presented 2–4 times with intervals of more than 1 min.

**TABLE 1:** Reagents used in this study.

Chemicals	Abbreviation	Source	Identifier
Octanal	C8al	Wako Chemicals	Cat#150-00053
n-Tricosane	n-C23	Tokyo Chemical Industry	Cat#T0568
n-Pentacosane	n-C25	Tokyo Chemical Industry	Cat#T0139
n-Hexacosane	n-C26	Sigma-Aldrich	Cat#241687
n-Heptacosane	n-C27	Tokyo Chemical Industry	Cat# H0017
n-Octacosane	n-C28	Sigma-Aldrich	Cat#O504
n-Nonacosane	n-C29	Tokyo Chemical Industry	Cat#N0167
(z)-9-Tricosene	9-C23	Tokyo Chemical Industry	Cat#T1242
(z)-9-Pentacosene	9-C25	Synthesized	Uebi et al., 2022
(z)-9-Hexacosene	9-C26	Synthesized	Uebi et al., 2022
(z)-7-Heptacosene	7-C27	Synthesized	Uebi et al., 2022
(z)-9-Heptacosene	9-C27	Synthesized	Uebi et al., 2022
(z)-7-Nonacosene	7-C27	Synthesized	Uebi et al., 2022
(z)-9-Nonacosene	9-C27	Synthesized	Uebi et al., 2022
5-Methylheptacosane	5-MeC27	Synthesized	Uebi et al., 2022
13-Methylheptacosane	13-MeC27	Synthesized	Uebi et al., 2022
7,15-Dimethylheptacosane	7,15-DiMeC27	Synthesized	Uebi et al., 2022
5,7,12-Trimethylpentacosane	5,7,12-TriMeC25	Synthesized	Uebi et al., 2022
5,7,12-Trimethylheptacosane	5,7,12-TriMeC27	Synthesized	Uebi et al., 2022

To deliver vaporized CHCs to an antenna, we attached three heaters to our olfactory stimulation apparatus for SSR (Tateishi et al., 2022). Fresh air taken from outdoors via a diaphragm pump was cleaned and dried by charcoal and silica-gel filters, respectively. The dried air was passed through a copper tube heated in 50°C water, and then divided into two streams, stream 1 and 2. Both streams were maintained at 1 L/min using flowmeters. Stream 1 carried the vaporized CHC by passing over the glass tube containing a given CHC heated in a heating block at 80°C. To deliver CHCs stably to antennae, the copper tube carrying the vaporized CHC was heated again by a handmade heater to 80°C. Stream 2 passed through a heated glass tube without CHC was constantly applied to the antenna. The final temperature of both streams was 50°C.

The timing of CHC stimulation was controlled by two solenoid valves and one handmade air-shutter controlled by a stimulator (SEN7203, Nihon Kohden, Tokyo, Japan). The air-shutter was located between the nozzle tip of stream 1 and the antenna. First, stream 1 containing a given CHC was started by opening a solenoid valve, but the air did not reach the antenna because the air-shutter was closed. After running stream 1 for 1 s, constant air stream (stream 2) was stopped by closing the other solenoid valve and the air-shutter was opened at the same time. The CHC presentation was stopped 2 s after opening the air-shutter and stream 2 started again. Thus, ants received the CHC stimulus for 2 s. The air around the preparation was continuously removed through a duct.

### Pharmacological experiment

To investigate the electrical coupling between dendrites of SNs in single basiconic sensilla (Takeichi et al., 2018; Uebi et al., 2022), we used the general gap junction blocker, carbenoxolone (CBX) (Miriyala et al., 2018). 3 μL of ant saline (control group) or 10 mM CBX solution (CBX group) was injected into the hemolymph of the ice-anesthetized ant. After incubation for 5–9 h at room temperature, ants were anesthetized again and attached to the acrylic plate for SSRs. In each recording, we extracted waveforms for 2.5 ms before and after the peak of a large amplitude spontaneous spike of a SN and detected all peaks of synchronized spikes within the 5-ms time window. Electrical events were acquired at a sampling rate of 20 kHz, and we defined the spike peak when the waveform potential was increased and decreased for three consecutive sampling points, respectively. In each recording, we counted the number of the large amplitude spontaneous spikes within the 3-s period of recording, and calculated the appearance ratio of the synchronized spikes.

### Spike sorting and analysis

**FIGURE 1:**
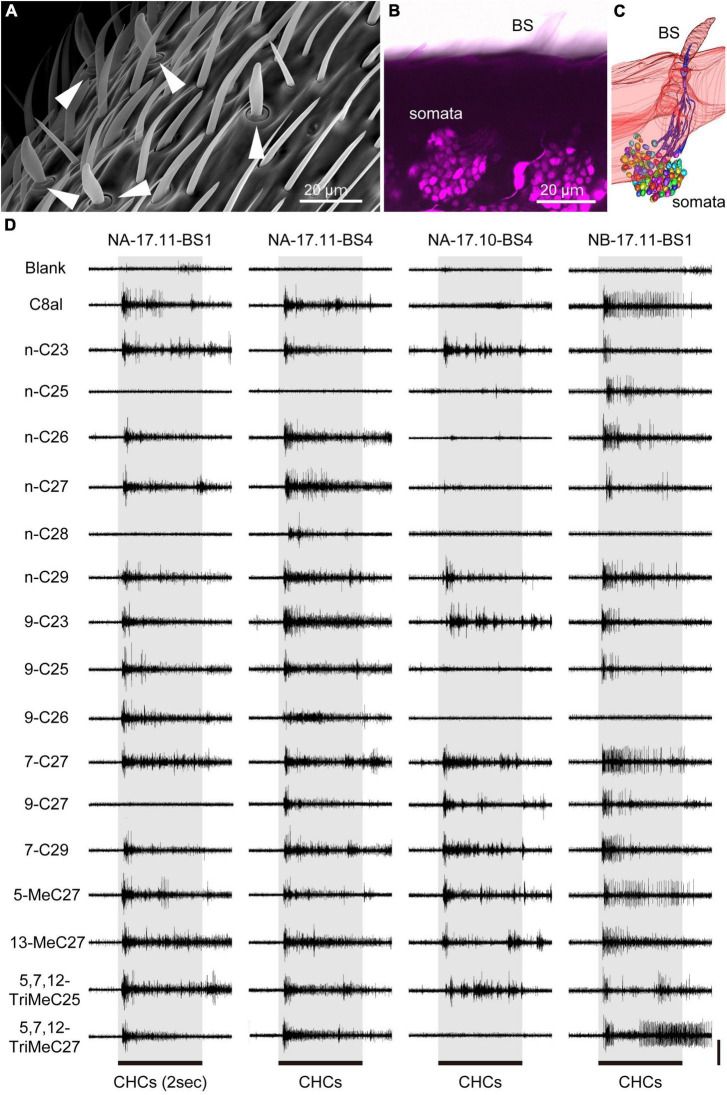
Extracellular recordings from single basiconic sensilla in *C. japonicus*. **(A)** Basiconic sensilla on the ant antenna. Basiconic sensilla are indicated by arrowheads. **(B,C)** SNs in a basiconic sensillum. Retrograde staining of antennal afferents **(B)** and their 3D reconstruction **(C)** revealed approximately 140 SNs in a single basiconic sensilla. **(D)** Responses of four basiconic sensilla to tested CHCs. Each basiconic sensillum exhibited “all-or-nothing” responses to tested CHCs. Recorded basiconic sensilla are termed by “colony name (NA, NB, or NC)–sampling date (year. month)–sensillum number (BS1-6)”. The 2-s CHC stimuli are indicated by gray boxes and horizontal bars under the electrophysiological traces. CHC responses of 23 sensilla used in this study are denoted in Supplementary Figures 3–5. Vertical bar = 0.2 mV.

There are approximately 140 SNs in a single basiconic sensillum of *C. japonicus* (Figures 1A–C: Nakanishi et al., 2009); therefore, spikes of several shapes were discharged from different SNs in a single sensillum and concurrently recorded. We sorted spikes of different shapes from several recordings using spike sorting software SpikeTaro (Chinou Jouhou Shisutemu Inc., Kyoto, Japan), which was developed so that even partially overlapping impulses can be recognized and sorted accordingly as different units. In another species of ant, *Formica yessensis*, the software successfully sorted the spikes of different SNs housed in single basiconic sensilla (Kidokoro-Kobayashi et al., 2012). The software sorted impulses based on two criteria; impulse amplitude and correlation coefficient of wave-forms. The impulses having amplitudes within ± 10% and wave-form coefficients over 0.85% were considered to be generated from the same SN. Recorded spikes with an appearance probability of less than 2% were omitted to remove unreliable impulse units. Because we extracted spikes whose amplitudes were over ∼0.05 mV, we could not detect small amplitude spikes. Nevertheless, we clearly discriminated 9–17 units in each single basiconic sensillum recording.

Cuticular hydrocarbon responses of both sensilla and SNs were evaluated as an increase in spike frequency from the spontaneous level; R-R_0_, where R and R_0_ were the numbers of spikes during the 2-s periods after and before the onset of CHC stimulation, respectively. In each recording, response intensity to a given CHC was represented as the averaged R-R_0_ of two to four repeating stimuli. To evaluate the CHC response at the sensillar level, we counted all spikes over an arbitrarily selected threshold and calculated the response intensities to CHCs. In the mass response, response intensities varied across individual sensilla, and the response intensities to CHCs were normalized against the maximum response intensity to a given CHC. The response intensities of each SN and relative response of each sensillum to tested CHCs are shown as heatmaps. To characterize response properties of recorded sensilla and SNs, we performed hierarchical cluster analysis using Ward’s method attached in R v.3.3.2 software (R Foundation for Statistical Computing, Vienna, Austria). Based on similarities of response spectra to tested CHCs, recorded sensilla and SNs were classified into several groups using bootstrap *P*-values. We considered that SNs and sensilla belonging to the clade whose bootstrap *P*-value was >0.90 exhibited similar CHC response spectra.

To compare combinatory activity patterns of SNs elicited by each CHC, we performed hierarchical cluster analysis and principal component analysis (PCA) across SNs using the “cluster” package in R software. Using the “clusplot” package in R software, we plotted scores of the first three principal components (PCs) obtained from PCA. To compare the CHC response spectra among different colonies, we performed PCA using the response spectra of 170 sorted SNs to 16 CHCs. Distributions of the first two PCs were summarized as box plots based on the colony identities and variances of PCs were statistically compared by one-way ANOVA and the *post hoc* Tukey test using R software.

## Results

### CHC responses of single basiconic sensilla

We collected worker castes of the Japanese carpenter ant *C. japonicus* from three different colonies (NA, NB, and NC). *C. japonicus* uses 18 CHCs for nestmate recognition, including C23–C29 alkanes, alkenes, and methyl-branched alkanes (Table 1; Ozaki et al., 2005). CHCs exhibit low-volatility at room temperature; therefore, a 1 mM CHC solution diluted in paraffin oil was vaporized at 80°C and delivered to antennae via a heated air (∼50°C). In our preliminary behavioral experiments, *C. japonicus* exhibited significant aggressive behaviors toward the vaporized non-nestmate CHCs heated by 50°C air carrier (Supplementary Figure 1), consistent with the behavioral study reported for another ant species, *C. floridanus* (Brandstaetter et al., 2008).

**FIGURE 2:**
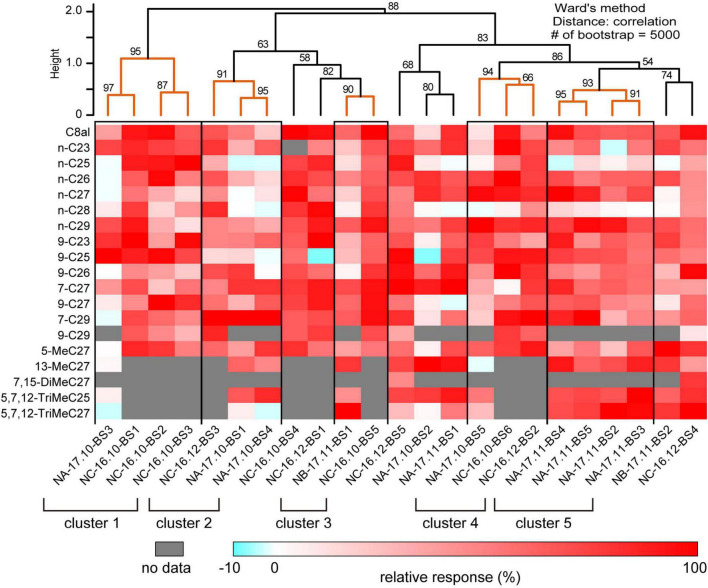
Cuticular hydrocarbon (CHC) response spectra of 23 basiconic sensilla. The CHC response spectra of 23 basiconic sensilla (Supplementary Figures 3–5) are shown as a heat map of relative response intensities. Based on the similarities of CHC response spectra, we performed cluster analysis using Ward’s method, and 23 basiconic sensilla were classified into several groups as shown in the dendrogram. The numbers denoted in the dendrogram indicate the bootstrap *P*-value (%). We considered that sensilla belonging to the clade whose bootstrap *P*-value was >0.90 exhibit similar CHC response spectra. In this study, we identified five groups of basiconic sensilla (clusters 1–5).

We succeeded SSRs from 23 basiconic sensilla from different ants belonging to one of five groups with analyzable signal-noise ratio (Supplementary Figures 3–5). In each recording, responses to more than 13 CHCs were attained. The 23 basiconic sensilla (BS) were designated as “colony name (NA, NB, or NC)–sampling time (year. month)–sensilla number (BS1-6).” A basiconic sensillum houses approximately 140 SNs (Figures 1A–C; Nakanishi et al., 2009, 2010). SSR recorded action potentials (spikes) of multiple amplitudes that must have originated from different SNs in a sensillum. Each recorded sensillum exhibited spontaneous spike activities. Effective CHCs elicited reproducible phasic-tonic spike responses that lasted for the duration of the stimulation period, while some CHCs did not elicit any response (Figure 1D; Supplementary Figures 3–5). Each sensillum, therefore, responded in an “all-or-nothing” manner to a given CHC (Figure 1D; Supplementary Figures 3–5). Different sensilla exhibited excitatory responses to different sets of CHCs (Figures 1D, 2; Supplementary Figures 3–5). CHC response spectra of 23 sensilla revealed that all sensilla were broadly tuned to CHCs (Figure 2) and that they could be grouped into five clusters (clusters 1–5 in Figure 2) based on response spectra similarities. Four sensilla of cluster 1 exhibited strong excitatory responses to the shorter chained alkanes and alkenes of up to C25, and three of four sensilla were from ants collected from colony NC in November 2016. Four sensilla of cluster 5 exhibited no responses to n-C25 and n-C26, and all four sensilla were from ants collected from colony NA in November 2017. These results indicate that basiconic sensilla of ants that were collected at the same time and from the same colony tend to have similar CHC response profiles.

### Responses to CHCs of sensory neurons co-localized in single basiconic sensilla

**FIGURE 3:**
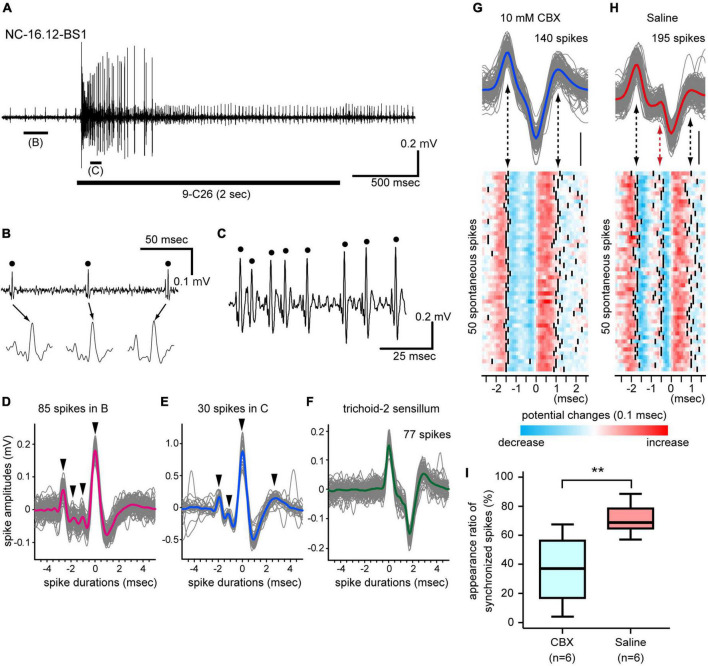
Synchronized firing of multiple SNs in single basiconic sensilla. **(A)** Response to 9-C26. A horizontal bar under the electrophysiological trace is the stimulus period. **(B,C)** Expanded electrophysiological traces shown in panel **(A)**. Some SNs in a basiconic sensillum exhibit spontaneous spike activity **(B)**, and the 9-C26 stimulus recruited a different set of SNs **(C)**. **(D,E)** Synchronized firing of multiple SNs. Traces of 85 spontaneous spikes [dots in panel **(B)**] and 30 CHC-induced spikes [dots in panel **(C)**] are superimposed in panels **(D,E)**, and averaged spike shapes are shown by red and blue lines, respectively. The largest spikes temporally synchronized with several small spikes (arrowheads). **(F)** Spike shapes of SNs in another olfactory sensillum. Traces of 77 spontaneous spikes of SNs in a trichoid-2 sensillum are superimposed, and averaged spike shapes are shown by a green line. The trichoid-2 sensillum houses 8–9 SNs, but the synchronization of spikes was not observed in the electrophysiological traces. **(G,H)** Effects of a general gap junction inhibitor on spike activities. Traces of 85 spontaneous spikes from a CBX-injected ant and 195 spikes from a saline injected ant are superimposed in panels **(G,H)**, and averaged spike shapes are shown as blue and red lines, respectively. Among these spontaneous spikes, 50 typical spikes were arbitrarily selected and are shown as heatmaps. The timing of spike peaks is denoted by solid lines (lower panels). In the saline-injected group, the synchronized spikes are denoted by a red arrow in panel **(H)**. **(I)** Appearance ratio of synchronized spikes. Spontaneous spike activities of six basiconic sensilla from 6 CBX-injected ants and six basiconic sensilla from 6 saline-injected ants were recorded. In each recording, the appearance ratio of the synchronized spikes was calculated using spontaneous spikes within the 3-s period of time window. The line in the box and the box represents the median and the quartiles, respectively. The result of the statistical comparison is denoted by asterisks (one-way ANOVA; *P* < 0.05).

The basiconic sensilla of *C. japonicus* exhibited excitatory responses to many kinds of CHCs, similarly to basiconic sensilla of other ants, e.g., *C. floridanus* (Sharma et al., 2015) and *Harpegnathos saltator* (Ghaninia et al., 2017). However, the physiological features and CHC response properties of individual SNs in single basiconic sensilla have not been assigned in any ants. Basiconic sensilla generally exhibited spontaneous spike activities. During the stimulation period, an effective CHC increased the spontaneous spike frequency and recruited many different SNs (Figure 3A; Supplementary Figures 3–5). In single basiconic sensilla, large spontaneous and CHC-induced spikes accompanied multiple small spikes, and these small spikes were temporally synchronized with the large spikes (Figures 3B–D; Supplementary Figures 3–5). Because spikes of different shapes were synchronized at different times between the non-stimulus and stimulus periods, different sets of SNs were assumed to be recruited between these periods (Figures 3D, E). Different basiconic sensilla exhibited different patterns of synchronized spikes (Supplementary Figures 3–5). This synchronization was not observed in spontaneous activities in another type of olfactory sensilla, such as trichoid-2 sensilla, which house 8–9 SNs (Figure 3F; Nakanishi et al., 2009). In *C. japonicus*, anatomical study revealed the dendrites of multiple SNs in single basiconic sensilla are connected by gap junctions (Takeichi et al., 2018). Therefore, we recorded spontaneous spike activities of SNs in single basiconic sensilla using ants injected with the general gap junction inhibitor, CBX, (CBX group) (Figure 3G). In ants injected with saline solution (control group: Figure 3H), peaks of small amplitude spikes synchronized with the large amplitude spontaneous spikes, and 71% of the large spontaneous spikes accompanied small amplitude spikes (Figure 3I). In the CBX group, the averaged appearance ratio of synchronized spikes was 36% and it was significantly smaller than that in the control group (Mann–Whitney *U*-test: *P* = 0.0087). Desynchronization of SN activities due to CBX, together with the ultramicroscopic study (Takeichi et al., 2018), strongly suggest that multiple SNs in a basiconic sensillum are electrically coupled.

**FIGURE 4:**
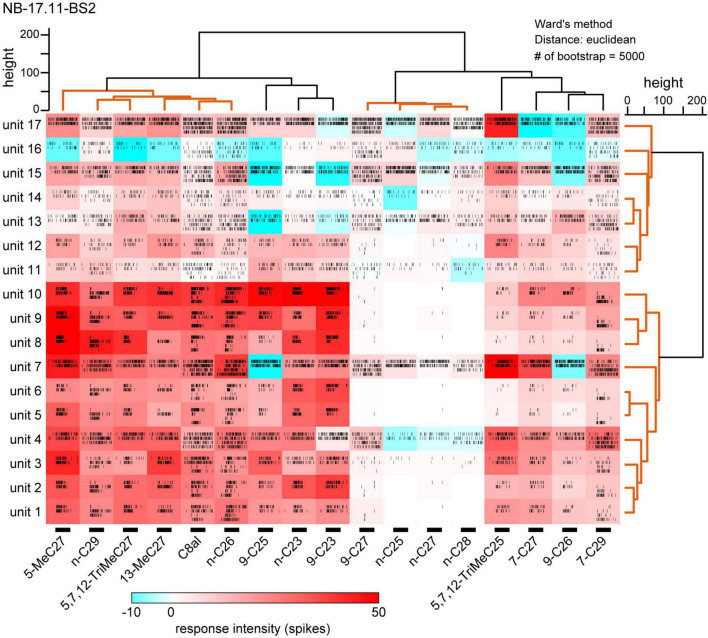
Cuticular hydrocarbon (CHC) response spectra of 17 sorted SNs in a basiconic sensillum. We sorted spike units of 17 SNs (units 1–17) from the multi-unit recording of a basiconic sensillum (NB-17.11-BS2). Response intensities of each sorted SNs to 16 tested CHCs are shown as a heatmap. Each CHC was presented to an antenna two to four times, and temporal activity patterns of each sorted SN are shown as raster plots. The bars under the heatmap indicate the 2-s period of CHC stimulation. Using cluster analyses, we clustered sorted SNs based on the similarities of response spectra (the right dendrogram), and clustered tested CHCs based on the spatial activity patterns of SNs (the upper dendrogram). According to the bootstrap *P*-values (>0.90), we identified several groups of CHCs and SNs (orange lines in dendrograms). CHC responses in each of 23 basiconic sensilla were analyzed using the same method, and results of spike sorting and cluster analysis of CHCs are summarized in Table 2.

Using software developed to discriminate simultaneously recorded single unit spikes from multiple unit spikes, we sorted spikes of individual SNs in single basiconic sensilla (Perez Goodwyn et al., 2009; Kidokoro-Kobayashi et al., 2012). Because the software detected spikes over above-threshold amplitudes (in this study ∼0.06 mV), we did not analyze unreliable, smaller amplitude spikes, which were synchronized with the large amplitude spikes (Figure 3). Nevertheless, we reliably discriminated 9–17 spike units from different SNs in each recording of single basiconic sensilla (Table 2). For example, we identified different spikes of 17 SNs in a recording from a sensillum (Figure 4; sample number: NB-17.11-BS2). Based on the similarity of response spectra to 16 tested CHCs, 17 SNs were classified into two groups (right dendrogram in Figure 4). SNs belonging to the same cluster represented similar temporal activity patterns to given CHCs (raster plots in Figure 4). Cluster analysis also revealed that tested CHCs were classified into several groups based on activation patterns of SNs within the sensillum (upper dendrogram in Figure 4; Table 2). For example, activity patterns of SNs in NB-17.11-BS2 sensillum elicited by three methyl-branched C27 alkanes were similar, but were different from those elicited by n-C27 alkanes or by C27 alkenes. Therefore, olfactory information from single basiconic sensilla have the potential to discriminate several groups of CHCs based on the combined activity patterns of SNs co-localized in a sensillum. The clustering pattern of CHCs was different between individual basiconic sensilla (Table 2). The ant might finely discriminate each CHC by integrating the activity pattern of multiple basiconic sensilla.

**TABLE 2:** Results of spike sorting and cluster analysis of CHCs in each of 23 basiconic sensilla.

Sample #	# of sorted SNs	C8al		n-C25		n-C27		n-C29		9-C25		7-C27		7-C29		5-MeC27		7,15-DiMeC27		5,7,12-TriMeC27	Figures
			**n-C23**		**n-C26**		**n-C28**		**9-C23**		**9-C26**		**9-C27**		**9-C29**		**13-MeC27**		**5,7,12-TriMeC25**		
NA-17.10-BS1	14	a	a	a	a	a	a	b	b	a	b	b	a	b	ND	a	b	ND	b	a	2,5,6
NA-17.10-BS2	14	a	b	a	b	b	a	b	a	a	b	b	a	a	ND	a	b	ND	b	a	2,5,6
NA-17.10-BS3	13	b	c	a	a	a	a	c	c	c	a	b	a	a	ND	a	a	ND	a	a	2,5,6
NA-17.10-BS4	12	b		a	b	b	a	b		a	a	c			ND	c		ND		a	1,2,5,6
NA-17.10-BS5	9		c	c	a	a	c	a	a	a	c	c	c	c	ND	b	c	ND	b	c	2,5,6
NA-17.11-BS1	11	b	b	a	b	b	a	b	b	b	b	b	a	b	ND	b	b	ND	b	b	1,2,5,6
NA-17.11-BS2	15		a	a		c	a			c	c	c	c	b	ND	b	d	ND	d		2,5,6
NA-17.11-BS3	13	c	c	a	c	c	a	c	c	c	c	c	c	c	ND	c	b	ND	b	b	2,5,6
NA-17.11-BS4	12	a	b	b	a	a	b	a	a	a	b	a	a	a	ND	b	a	ND	a	a	1,2,5,6
NA-17.11-BS5	14	b	b	a	b	b	a	b	b	b	b	b	b	b	ND	b	b	ND	b	b	2,5,6
NB-17.11-BS1	12	c	b	b	d	b	b	c	a	a	b	c	d	d	ND	a		ND	d		1,2,5,6
NB-17.11-BS2	17	a		b	a	b	b	a					b		ND	a	a	ND		a	2,4,5,6
NC-16.10-BS1	15						b	c			a	b	a	b	b	c	ND	ND	ND	ND	2,5
NC-16.10-BS2	10					a		a	a		a						ND	ND	ND	ND	2,5
NC-16.10-BS3	14	a			c	b	c	b		a	b	c		c	c	c	ND	ND	ND	ND	2,5
NC-16.10-BS4	11		ND		c		b	d	d	a	d	b	b	a		c	ND	ND	ND	ND	2,5
NC-16.10-BS5	15	a	b	b	b	b	b	a	b	b	b	a	a	a	b	b	ND	ND	ND	ND	2,5
NC-16.10-BS6	14	b	a	d	a	b	c	b		a	a	c	d	a			ND	ND	ND	ND	2,5
NC-16.12-BS1	15	b	a	b	a	a	b	b	b		a	b	b	a	b	a	ND	ND	ND	ND	2,3,5
NC-16.12-BS2	13	a			b		a					b	b		b		ND	ND	ND	ND	2,5
NC-16.12-BS3	13	b	b		b	a	b	a	a		b	a	a	b	b	b	ND	ND	ND	ND	2,5
NC-16.12-BS4	12		a	a		a	a	a	a	a	a	a	a	a			a	a			2,5,6
NC-16.12-BS5	13		b	a	b				b	a	a	a		b		b	b		b		2,5,6

**FIGURE 5:**
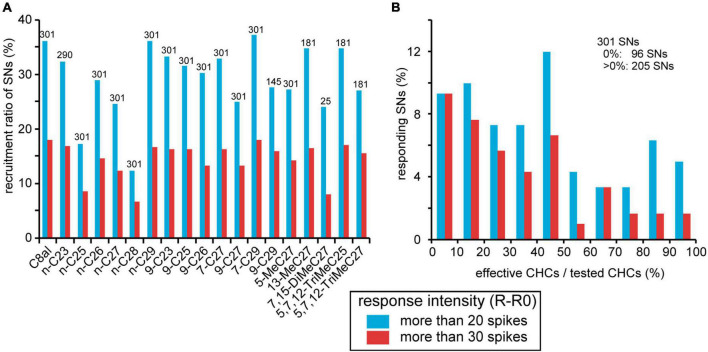
Summary of responses of 301 sorted SNs to CHCs. **(A)** Recruitment rates of SNs. CHC responses of 301 SNs from 23 sensilla were evaluated according to an increase in spike frequency from the spontaneous level (R-R0; See section “Materials and methods”). When the value was >20 spikes, we regarded the SN to be activated by the CHC. The percentages of activated SNs per CHC are shown as recruitment rates. Because it is not that all 301 sorted SNs were tested all 18 CHCs (Table 2), numbers above bars show the numbers of sorted SNs that we successfully recorded the response to the given CHC. **(B)** CHC specificities of SNs. Among 301 sorted SNs, 205 exhibited excitatory responses (R-R0 > 20 spikes) to more than one CHC. In each sorted SN, we calculated the percentages of the number of effective CHCs per that the number of tested CHCs as CHC specificity. Distribution of CHC specificities of 301 sorted CHCs is shown as a histogram. The percentage of SNs that did not respond to any tested CHC is omitted from the histogram. In both panels **(A,B)**, the response intensity of each SN to a given CHC is represented by two levels of response intensity (R-R0 > 20, blue bars, R-R0 > 30, red bars).

**FIGURE 6:**
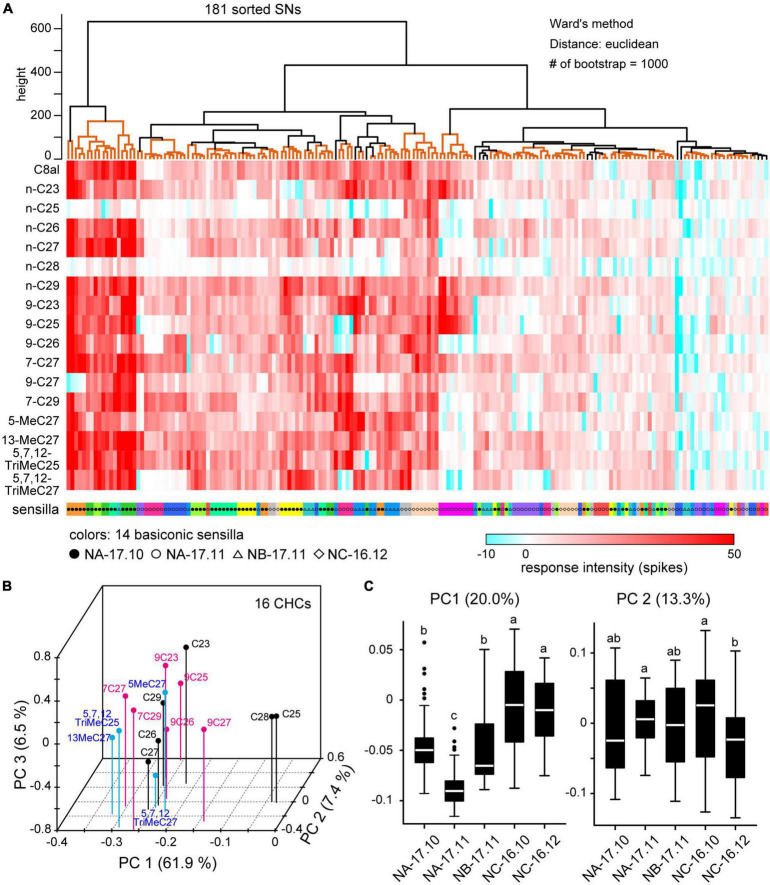
Cuticular hydrocarbon (CHC) coding of SNs in basiconic sensilla. **(A)** Response spectra of 181 SNs from 14 basiconic sensilla to 16 CHCs. CHC response spectra of 181 sorted SNs are shown in the heat map. Using cluster analysis and bootstrap *P*-values (>0.90), we grouped SNs based on the similarities of response spectra (clades denoted by orange lines in the upper dendrogram). SNs co-localized in the same sensillum are coded by the same color under the heat map. Black and white circles, triangles and diamonds denoted under the heat map show the colony identities. **(B)** The receptive space of CHCs. Based on the spatial activity patterns of SNs elicited by each CHC, we performed principal component analysis (PCA). The first three principal components (PCs) are mapped into a 3D receptive space. CHCs that elicited similar spatial activity patterns in SNs mapped closer together. CHCs of alkanes, alkenes and methyl-branched alkanes are differentially color-coded. **(C)** CHC response spectra for nestmate recognition. Using CHC response spectra of SNs, we performed PCA. The distributions of the first two PCs are summarized based on the colony information, and are displayed as box plots. The line in the box and the box represents the median and the quartiles, respectively. Outliers are shown as dots. The results of multiple comparisons are denoted as different letters on the box plots (one-way ANOVA; *P* < 0.05).

The number of sorted sensilla in each of 23 sensilla are summarized. In each recording, we clustered tested CHCs based on the spatial activity patterns of SNs, and CHCs denoted the same alphabetical letters activate similar sets of SNs in single basiconic sensilla (see the upper dendrogram in Figure 4). The sample used in the analysis shown in Figures 1–6 are also denoted in the right column. ND, no data.

### Sensory coding of CHCs

We sorted CHC responses of 301 SNs from 23 basiconic sensilla throughout the experiments (Table 2). The increase in spike frequency during the CHC stimulus period from that of pre-stimulus period was defined as the response intensity of each SN. When the response intensity was >20 spikes, we judged the SN to be activated by the CHC. Based on the response intensities of 301 recorded SNs to CHCs, we estimated the recruitment ratio of SNs to each CHC and specificities of SNs (Figure 5). All CHCs other than the two alkanes, n-C25 and n-C28, activated more than 25% of SNs regardless of their chemical structures (Figure 5A). Among 301 SNs, 96 (31%) did not respond to any tested CHCs. Among the remaining 205 SNs (69%), 125 (42%) were excited by more than five different CHCs (Figure 5B). Thus, many SNs in basiconic sensilla of *C. japonicus* were broadly tuned to multiple CHCs.

Among 301 sorted SNs, we quantitatively analyzed responses of 181 SNs housed in 14 sensilla in which we successfully recorded the responses to 16 CHCs (Table 2; Figure 6). As shown in the heat map, each of 16 CHCs activated a large number of SNs with different combinatory patterns (Figure 6A). Using PCA, we constructed a 3-dimensional receptive space represented by the top three principal components (Figure 6B). In the receptive space, CHCs that activated similar combinatory patterns of SNs mapped close to one another, indicating similar perceptual qualities. Among the 16 CHCs, n-C28 and n-C25 were separated from the other CHCs and mapped closer together (Figure 6B), because they strongly activated specific subsets of SNs (Figure 6A). Except for the two alkenes, each CHC loosely clustered in the receptive space. Interestingly, alkenes (black dots), alkanes (magenta dots) and methyl-branched alkenes (blue dots), which belong to three different chemical groups, were separated in the receptive space. In addition, alkenes with a double-bond at C7–C8 were distinct from those with a double-bond at C9–C10. However, the shorter and longer chain CHCs were mapped in a jumble in the receptive space. These results indicated that peripheral sensory system of the ant has the ability to discriminate CHC chemical structures, but discriminative power of CHCs with different carbon chain lengths may be weak.

Using cluster analysis, we classified 181 SNs into various groups based on similarities of CHC response spectra (Figure 6A). The resulting cluster dendrogram (Figure 6A) revealed that SNs in a given sensillum tended to exhibit similar CHC response spectra. Nevertheless, several cluster groups were composed of SNs in different sensilla. In addition, SNs of different ants from the same colony tended to exhibit similar response spectra. To evaluate whether nestmate recognition is based on colony-specific CHC response properties of SNs, we performed PCA using CHC response spectra and summarized the first two principal components (PC1 and PC2) based on five sampling groups of ants (Figure 6C). PC1 variances of SNs in NA-17.11 and NB-17.11 ants were significantly different (ANOVA and *post hoc* Tukey’s test; *P* = 1.03 × 10^–5^), indicating that SNs of ants collected from different colonies exhibited different CHC response spectra. In addition, SNs of ants collected from the same colony on different time also exhibited different CHC response spectra (PC1 variances between NA-17.10 and NA-17.11 groups: *P* < 10^–7^, PC2 variances between NC-16.10 and NC-16.12 groups: *P* = 2.17 × 10^–4^). These results indicate that CHC response properties of SNs continuously change within the same colony.

## Discussion

In ants, to understand the neural mechanisms underlying the nestmate recognition, CHC receptive process have been analyzed based on the mass responses of single basiconic sensilla to CHC blends or the limited set of pure CHCs (Ozaki et al., 2005; Kidokoro-Kobayashi et al., 2012; Sharma et al., 2015; Ghaninia et al., 2017). In this study, using synthesized 18 CHCs that a *C. japonicus* uses for nestmate recognition, we assigned the CHC responsibilities not only of single basiconic sensilla but also of individual SNs co-housed in the sensillum for the first time. We recorded many simultaneous amplitude spikes that might have originated from different SNs co-localized in a single sensillum. Our recordings of 23 basiconic sensilla from different ants revealed that approximately 15 spikes were clearly distinguishable from thermal noise and each of them was synchronized with several small spikes. The synchronized spike activities and broadly tuned CHC response spectra of SNs strongly suggests that the gap junctions reported by the previous anatomical study are functionally connecting SN dendrites within a basiconic sensillum (Takeichi et al., 2018). In *C. japonicus*, there are approximately 180 basiconic sensilla are distributed on an antenna, and each sensillum housed about 140 SNs (Nakanishi et al., 2009). Therefore, we characterized CHC responses from a small portion of SNs from a small portion of basiconic sensilla. However, our study reveals important physiological features of SNs for CHC reception in the ant, and it furthers understanding of the peripheral mechanisms of nestmate recognition.

CHC responses of SNs in 23 basiconic sensilla revealed that most SNs in the sensilla exhibited broad CHC response spectra, and different CHCs activated different sets of SNs. In single sensillum, combinatory activity patterns of SNs revealed that CHCs were roughly classified into a few groups (Figure 4; Table 2), suggesting that the CHC discriminatory power of each basiconic sensillum is weak. However, the grouping patterns of CHCs varied across individual basiconic sensilla (Table 2). Our quantitative analysis revealed that *C. japonicus* has the potential to discriminate chemical structures of CHCs, such as long-chained alkanes, alkenes and methyl-branched alkanes, by integrating the combinatory activity pattern of SNs from multiple basiconic sensilla. In addition, among structurally related CHCs, *C. japonicus* might discriminate alkenes that have a double-bond at different positions. The result correlated with *C. japonicus* behavior; the ants exhibit remarkable aversive behavior to alkenes with a double-bond at C9–C10, but not to those with a double-bond at C7–C8 (Uebi et al., 2022). Interestingly, C28 and C25 long-chained alkanes are mapped to a region of the receptive space that was distinct from the other CHCs, indicating that these two CHCs have specific perceptual qualities. In the analysis of response spectra of CHC receptors in *H. saltator*, a CHC, 13,23-DiMeC37, was also separately mapped in the CHC receptive space (Pask et al., 2017). 13,23-DiMeC37 is received by a specific receptor and acts as a queen pheromone to mediate reproductive division within a colony. Therefore, further studies are needed to reveal behavioral functions of C28 and C25 long-chained alkanes in *C. japonicus*.

Multiple SNs in single basiconic sensilla fired synchronously in both spontaneous and CHC stimulus periods. The large identical amplitude spikes were generally accompanied by small amplitude spikes. We cannot exclude the possibility that we respectively recorded dendrite and axonal spikes of a cognate SN, which has been reported in electrophysiological recordings from insect auditory SNs (Warren and Matheson, 2018). Nevertheless, there are gap junctions between multiple dendrites in single basiconic sensilla of *C. japonicus* (Takeichi et al., 2018), and the general gap junction inhibitor, CBX, significantly impaired the spike synchronization. It is highly likely that synchronized firing of SNs results from the electrical coupling of multiple SNs via electrical synapses as reported in gustatory neurons of the bumble bee (Miriyala et al., 2018).

Our prominent finding in this study is that multiple SNs in single sensilla are broadly tuned to CHCs and exhibit similar CHC response spectra. The result is in contrast to CHC response spectra of each CHC receptor expressed in SNs of basiconic sensilla of another ant species *H. saltator* (Pask et al., 2017; Slone et al., 2017). In *H. saltator*, CHC response spectra of individual SNs have not assigned, but recordings from *Drosophila melanogaster* olfactory SNs, in which ant CHC receptors were heterologously expressed, revealed that each CHC receptor was very narrowly tuned to a few CHC ligands (Pask et al., 2017; Slone et al., 2017). The CHC receptor families have been identified in many ant species including *C. floridanus*, which is phylogenetically related to *C. japonicus* (Engsontia et al., 2015). It is still unknown response spectra of CHC receptors in *C. japonicus*. However, if the “one receptor–one SN” principle proposed in general insect is applicable in the SNs in basiconic sensilla of *C. japonicus*, each SN in basiconic sensillum may be narrowly tuned to a few CHCs according to the expressing CHC receptor. In contrast to them, we revealed in *C. japonicus* that SNs in a basiconic sensillum are more broadly tuned to CHCs. This could be explained by the micro-network functionally connecting SNs within a basiconic sensillum (Takeichi et al., 2018); each SN might acquire responsiveness to a broad range of CHCs by connecting dendrites of multiple SNs that express different CHC receptors via electric synapses.

In this study, we presented each CHC at a high concentration, in which elicit sufficient excitatory responses in SNs (Supplementary Figure 2). However, SNs in single basiconic sensilla responded to each CHC in an “all-or-nothing” manner (Figures 1, 2; Supplementary Figures 3–5). That is, effective CHCs elicited strong responses regardless of the kind of CHC, whereas ineffective CHCs did not elicit any spike activity. In addition, nestmate ants reared together in a group shared the effective and ineffective CHCs (e.g., C25 and C28 in NA-17.11), indicating that CHC reception is affected by the nestmate CHC blend that the ant carries. This interesting physiological feature can be explained by the pre-filter hypothesis (Ozaki et al., 2005), and by “stronger stimulus inputs spread” and “weaker stimulus inputs cut” filter functions deduced by mathematical modeling of gap junctions in *C. japonicus* basiconic sensillum (Takeichi et al., 2018). CHCs, which are abundantly distributed on the body surface, might elicit weak responses in SNs through sensory adaptation, and the weak responses of SNs might be eliminated by the “weaker stimulus inputs cut” function of gap junctions. In fact, prevention of antenna-cleaning (grooming) induced the accumulation of antennal CHCs and impaired the behavioral discrimination of nestmate and non-nestmate CHC blends in *C. japonicus* (Mizutani et al., 2021). In contrast, salient CHCs, such as CHC pheromones, effectively activated multiple SNs via the “stronger inputs spread” filter function. This corresponds with a high concentration of 9-tricosene (9-C23) activating multiple OSNs in single basiconic sensilla and indiscriminately activating many targeted T6 glomeruli in the antennal lobe of *C. japonicus* (Uebi et al., 2022). Thus, two peripheral sensory filters formed by sensory adaptation and gap junctions may emphasize the CHC information as the “all-or-nothing” response. The “all-or-nothing” CHC responses of basiconic sensilla are likely observed in the other *Camponotus* ant (Sharma et al., 2015). Therefore, it needs to investigate whether the “all-or-nothing” CHC responses and micro-network in the sensillum are common physiological and anatomical features in ant basiconic sensilla.

In ants collected from the same colony and reared together in a group, the CHC response spectra of SNs were similar at both sensillar and single SN levels. However, ants collected from different colonies tended to exhibit different CHC response spectra. Ants collected on different dates, even from the same colony, exhibited different CHC response spectra. The group-housed ants share a given CHC blend via trophallaxis and cuticular contacts and the compositional ratio of the CHC blend is continuously updated (Bos and d’Ettorre, 2012). Therefore, separately reared ants carry different CHC blends and exhibit aggressive behaviors toward each other (Vander Meer et al., 1989; Lahav et al., 2001). Because CHC responses of SNs in basiconic sensilla are strongly affected by the chronic exposure of nestmate CHC blends of *C. japonicus* (Ozaki et al., 2005), the different CHC response spectra of SNs between different ant groups may originate from CHC blend within the group.

Taken together our results with previous results, we hypothesize neural mechanisms underlying nestmate recognition in *C. japonicus*. In *C. japonicus*, SNs do not respond to the contact stimulus of nestmate CHC blend because these SNs have been adapted (Ozaki et al., 2005). However, we found that *C. japonicus* can discriminate colony membership using the vaporized CHC blend, as has been suggested for other ant species (Supplementary Figure 1; Brandstaetter et al., 2008). Because the volatility of each CHC is different, the compositional ratio of a vaporized nestmate CHC blend is different from the CHC blend carried by the ant. These results indicate that *C. japonicus* can discriminate colony membership based on the activity patterns of SNs elicited by the CHC information that passes the pre-filter adaptation. In other ant species, the vaporized nestmate CHC blend activates multiple SNs in a given basiconic sensillum (Sharma et al., 2015) and is represented in the female-specific glomeruli of the antennal lobe (Brandstaetter and Kleineidam, 2011; Brandstaetter et al., 2011). Thus, in each basiconic sensillum of *C. japonicus*, CHCs are first processed by two peripheral sensory filters, sensory adaptation and gap junctions, and are roughly coded as combinatory activity patterns of SNs. Then, by integrating CHC responses from multiple basiconic sensilla, CHC information is represented in detail as spatial activity patterns and response intensities of specific T6 glomeruli in the antennal lobe (Nakanishi et al., 2010; Stroeymeyt et al., 2010; McKenzie et al., 2016). Finally, spatial activity patterns of T6 glomeruli may be deciphered in specific regions of the higher brain centers, and be used for nestmate and caste recognitions (Nishikawa et al., 2012; Neupert et al., 2018). Thus, *C. japonicus* might utilize both the “pre-filtering” and the “template matching” for the discrimination of CHC blends. The “pre-filter hypothesis” is a suitable for the quick and reliable discrimination of significant differences of CHC blends between the nestmate and non-nestmate, and the “template matching hypothesis” is a suitable for the subtle differences of CHC blends within the colony, such as caste discrimination.

## Data availability statement

The raw data supporting the conclusions of this article will be made available by the authors, without undue reservation.

## Author contributions

HW and FY designed the experiments. SO, NN, KT, and HN performed the experiments. MO and RM synthesized the CHCs used in this study. HW, SO, NN, and KT analyzed the data. HW wrote the manuscript. MO and FY supervised the study. All authors had full access to all the data in the study and take responsibility for the integrity of the data and the accuracy of the data analysis.

## Supplementary material

Supplementary Figure 1 Aggressive behaviors to vaporized non-nestmate CHC extract. **(A)** Temperature dependent changes of behavioral responses to the non-nestmate CHC extract. Using the CHC delivery system, we observed behavioral responses of the tethered ants to the non-nestmate CHC extract heated by the air carrier to the given temperature. The tethered ants exhibited the stronger aggressive behaviors to the non-nestmate CHCs heated above 50°C (Wilcoxon signed-rank test; NS > *P* = 0.05, * < *P* = 0.05). **(B)** Aggressive behaviors to the nestmate and non-nestmate CHC extracts vaporized by a 50°C air carrier. Ants exhibited stronger aggressive behaviors to the vaporized non-nestmate CHC extract than to the vaporized nestmate CHC extract (chi-square test; *P* = 0.0027; ^**^ < *P* = 0.01). We determined levels of aggressiveness of the tethered ants according to previous studies as follows; 0: no responses, 1: intense antennal scanning during the stimulus period, 2: mandibles slightly opened, 3: mandibles widely opened, 4: body jerking with open mandibles, 5: gaster twisted forward to spray formic acid (Hölldobler and Wilson, 1990; Brandstaetter et al., 2008). When the ant exhibited 3, 4, or 5 behavioral responses during the recording period, we regarded that the ant exhibited the aggressive behavior to the vaporized CHC extract (dotted lines).

Supplementary Figure 2 Responses of SNs in single basiconic sensilla to a given concentration of 9-tricosene. **(A)** Responses of a basiconic sensillum to a given concentration of 9-tricosene (9-C23). Basiconic sensilla exhibited dose-dependent responses to 9-C23. The 2-s 9-C23 stimuli are indicated by horizontal bars under the electrophysiology traces and gray boxes. **(B)** Responses of sorted SNs to a given concentration of 9-C23. Responses of 65 sorted SNs from five basiconic sensilla are shown in the heat map. Using cluster analysis and bootstrap *P*-values (>0.90), we identified four groups (clusters 1–4) in which SNs exhibited similar dose-responses (clades denoted by magenta lines in the upper dendrogram). SNs co-localized in the same sensillum are coded by the same color under the heat map. **(C)** Dose-response curves of SNs. In each of the four groups identified in panel **(B)** (clusters 1–4), response intensities of SNs to a given concentration of 9-C23 are plotted as means ± SEMs. In each group, response intensities to different concentrations of 9-C23 are compared (one-way ANOVA and *post hoc* Tukey’s test), and the same letters represent no statistically significant differences (*P* > 0.05).

Supplementary Figures 3–5 Cuticular hydrocarbon (CHC) responses of 23 basiconic sensilla analyzed in this study. Each basiconic sensillum exhibited “all-or-nothing” responses to tested CHCs. In each sensillum, each of large spontaneous spikes are temporally synchronized with several small spikes. Recorded basiconic sensilla are termed by “colony name (NA, NB, or NC)–sampling date (year. month)–sensillum number (BS1-6).” The 2-s CHC stimuli are indicated by gray boxes and horizontal bars under the electrophysiological traces.
