# Supplementary figures and images for: Cuticular hydrocarbon reception by sensory neurons in basiconic sensilla of the Japanese carpenter ant
Source: Front Cell Neurosci. 2023 Feb 6;17:1084803. doi: 10.3389/fncel.2023.1084803 (PMC9940637; doi:10.3389/fncel.2023.1084803)

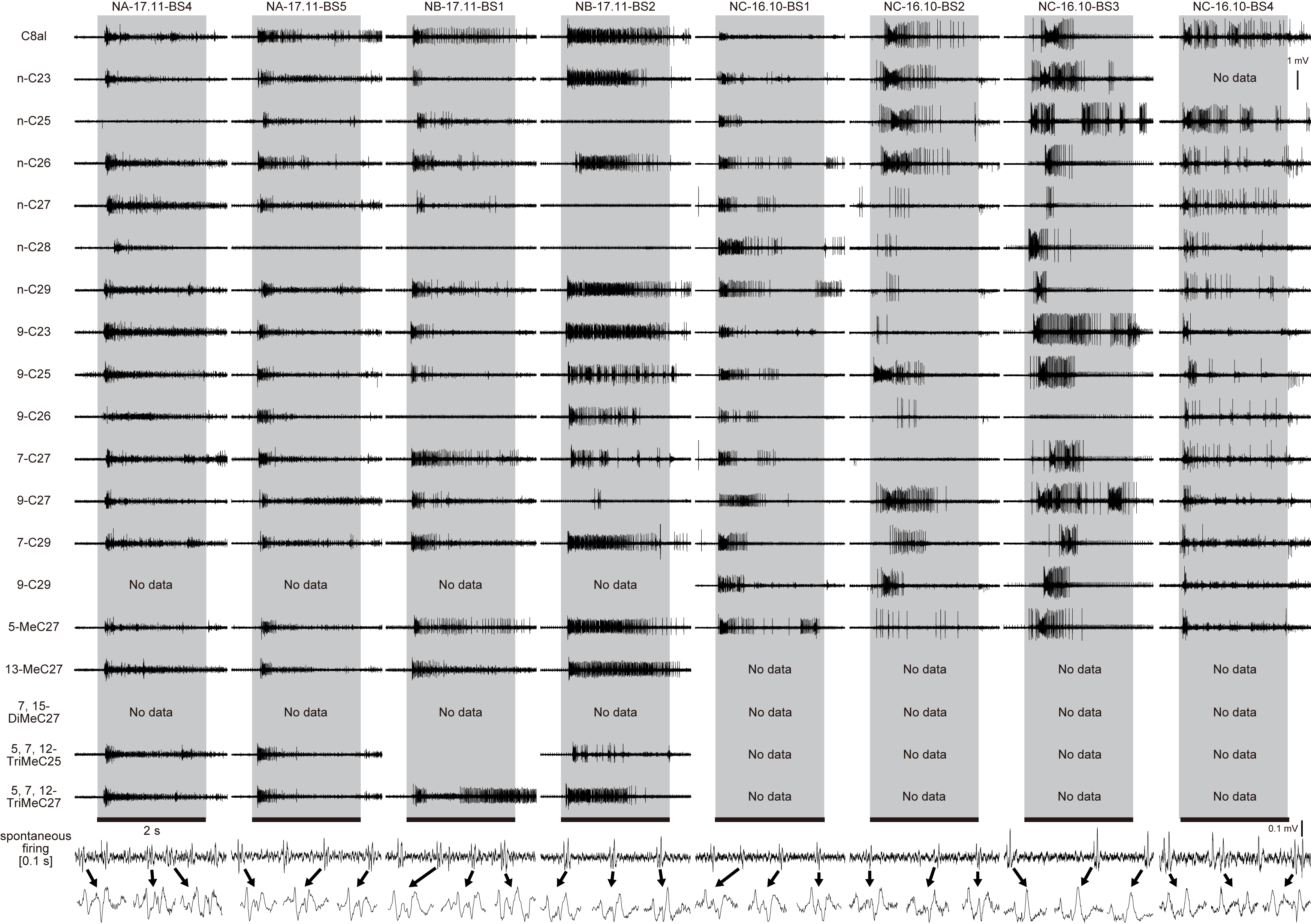

Supplement: Supplementary file 1 [file Image_4.JPEG]

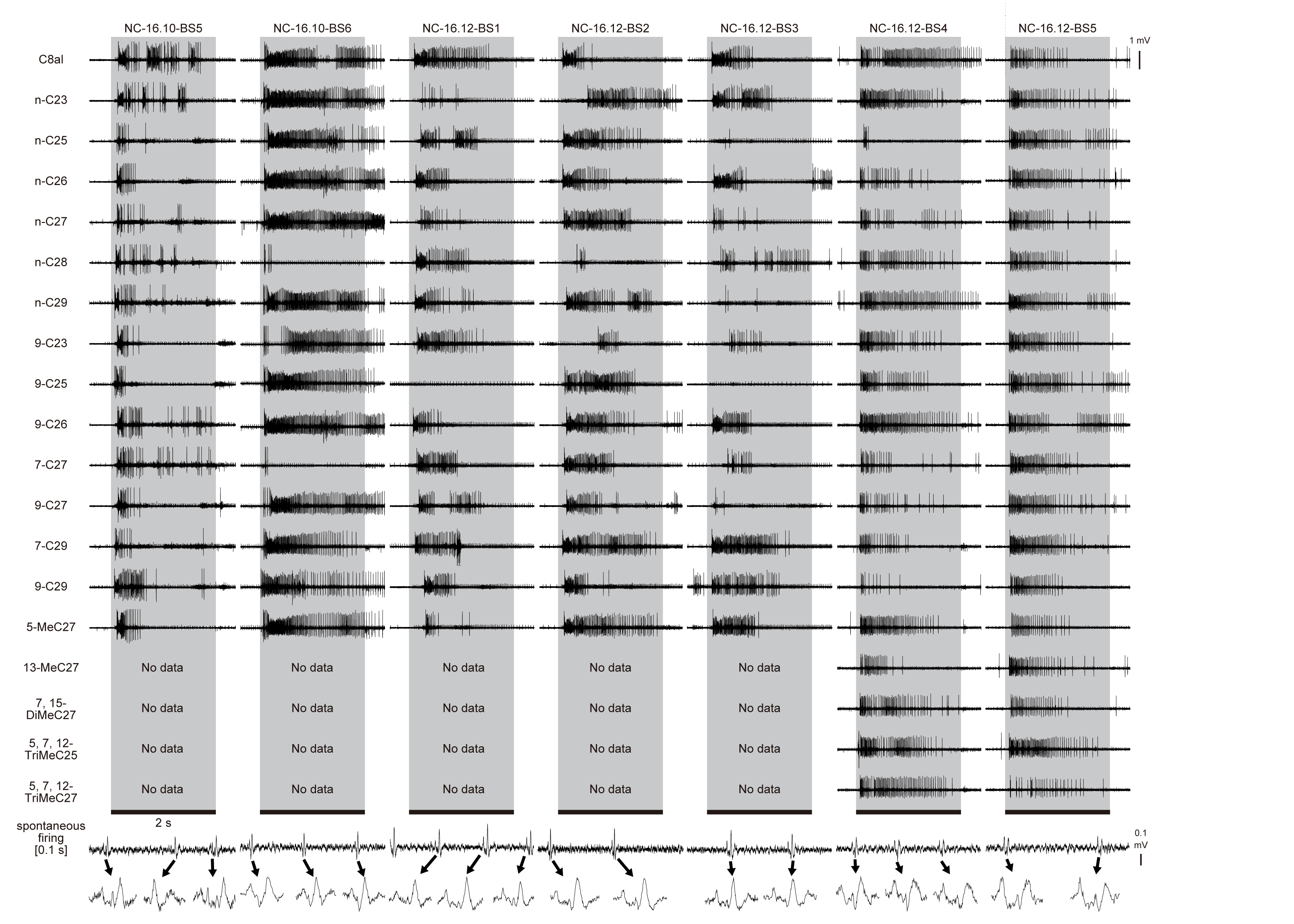

Supplement: Supplementary file 2 [file Image_5.JPEG]

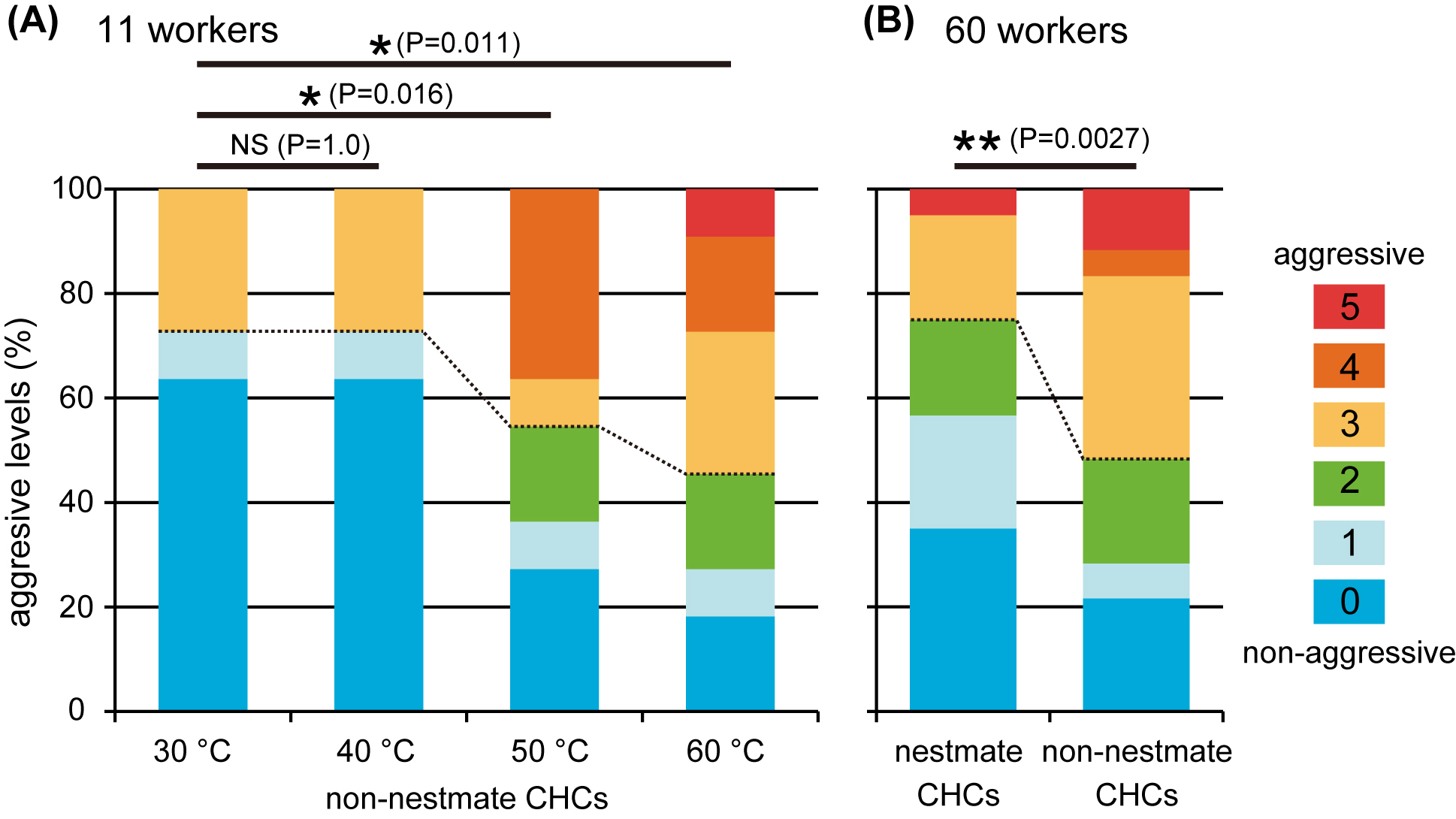

Supplement: Supplementary Figure 1 — Aggressive behaviors to vaporized non-nestmate CHC extract. (A) Temperature dependent changes of behavioral responses to the non-nestmate CHC extract. Using the CHC delivery system, we observed behavioral responses of the tethered ants to the non-nestmate CHC extract heated by the air carrier to the given temperature. The tethered ants exhibited the stronger aggressive behaviors to the non-nestmate CHCs heated above 50°C (Wilcoxon signed-rank test; NS > P = 0.05, * < P = 0.05). (B) Aggressive behaviors to the nestmate and non-nestmate CHC extracts vaporized by a 50°C air carrier. Ants exhibited stronger aggressive behaviors to the vaporized non-nestmate CHC extract than to the vaporized nestmate CHC extract (chi-square test; P = 0.0027; ** < P = 0.01). We determined levels of aggressiveness of the tethered ants according to previous studies as follows; 0: no responses, 1: intense antennal scanning during the stimulus period, 2: mandibles slightly opened, 3: mandibles widely opened, 4: body jerking with open mandibles, 5: gaster twisted forward to spray formic acid (Hölldobler and Wilson, 1990; Brandstaetter et al., 2008). When the ant exhibited 3, 4, or 5 behavioral responses during the recording period, we regarded that the ant exhibited the aggressive behavior to the vaporized CHC extract (dotted lines). [file Image_1.JPEG]

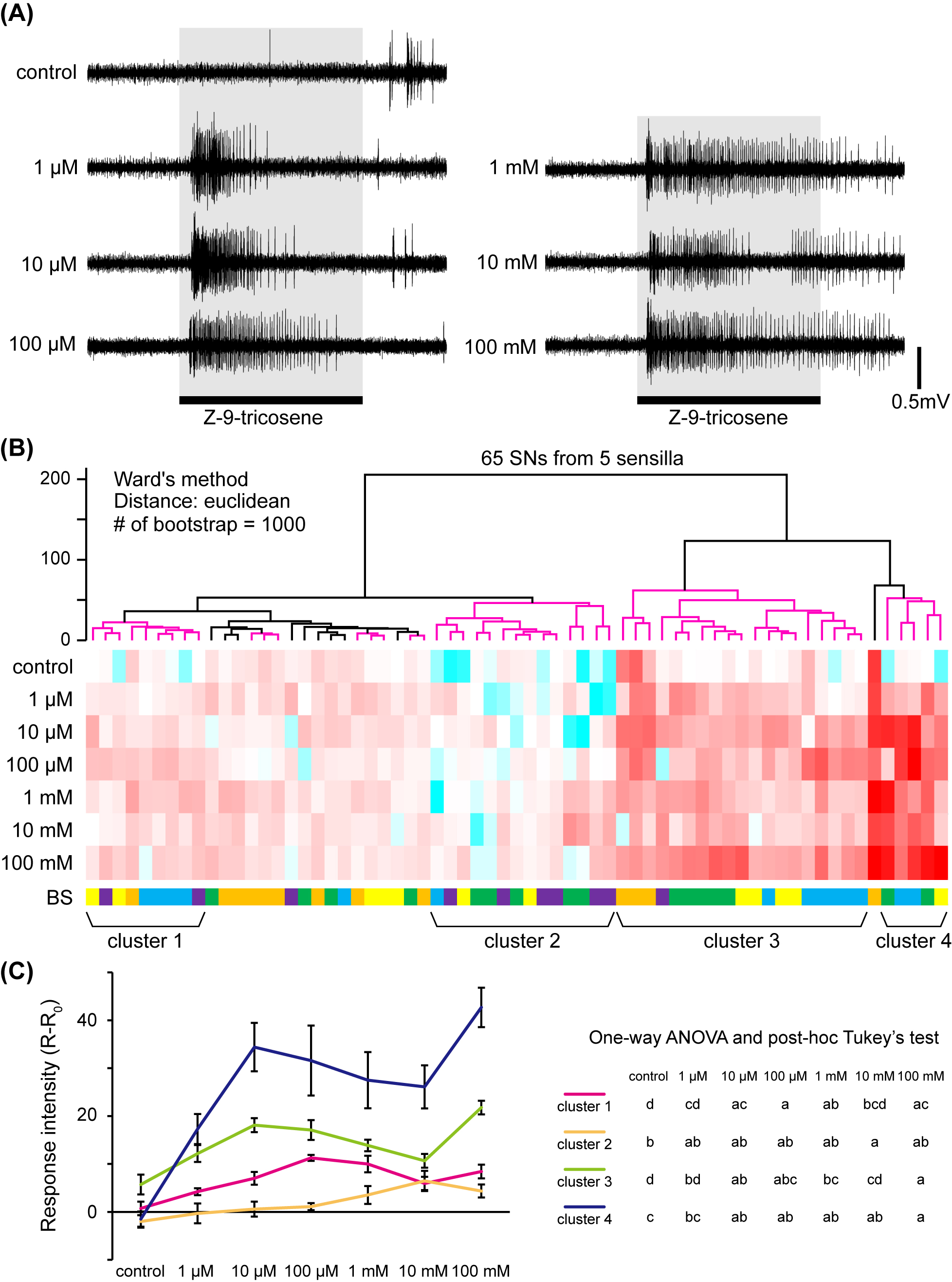

Supplement: Supplementary Figure 2 — Responses of SNs in single basiconic sensilla to a given concentration of 9-tricosene. (A) Responses of a basiconic sensillum to a given concentration of 9-tricosene (9-C23). Basiconic sensilla exhibited dose-dependent responses to 9-C23. The 2-s 9-C23 stimuli are indicated by horizontal bars under the electrophysiology traces and gray boxes. (B) Responses of sorted SNs to a given concentration of 9-C23. Responses of 65 sorted SNs from five basiconic sensilla are shown in the heat map. Using cluster analysis and bootstrap P-values (>0.90), we identified four groups (clusters 1–4) in which SNs exhibited similar dose-responses (clades denoted by magenta lines in the upper dendrogram). SNs co-localized in the same sensillum are coded by the same color under the heat map. (C) Dose-response curves of SNs. In each of the four groups identified in panel (B) (clusters 1–4), response intensities of SNs to a given concentration of 9-C23 are plotted as means ± SEMs. In each group, response intensities to different concentrations of 9-C23 are compared (one-way ANOVA and post hoc Tukey’s test), and the same letters represent no statistically significant differences (P > 0.05). [file Image_2.JPEG]

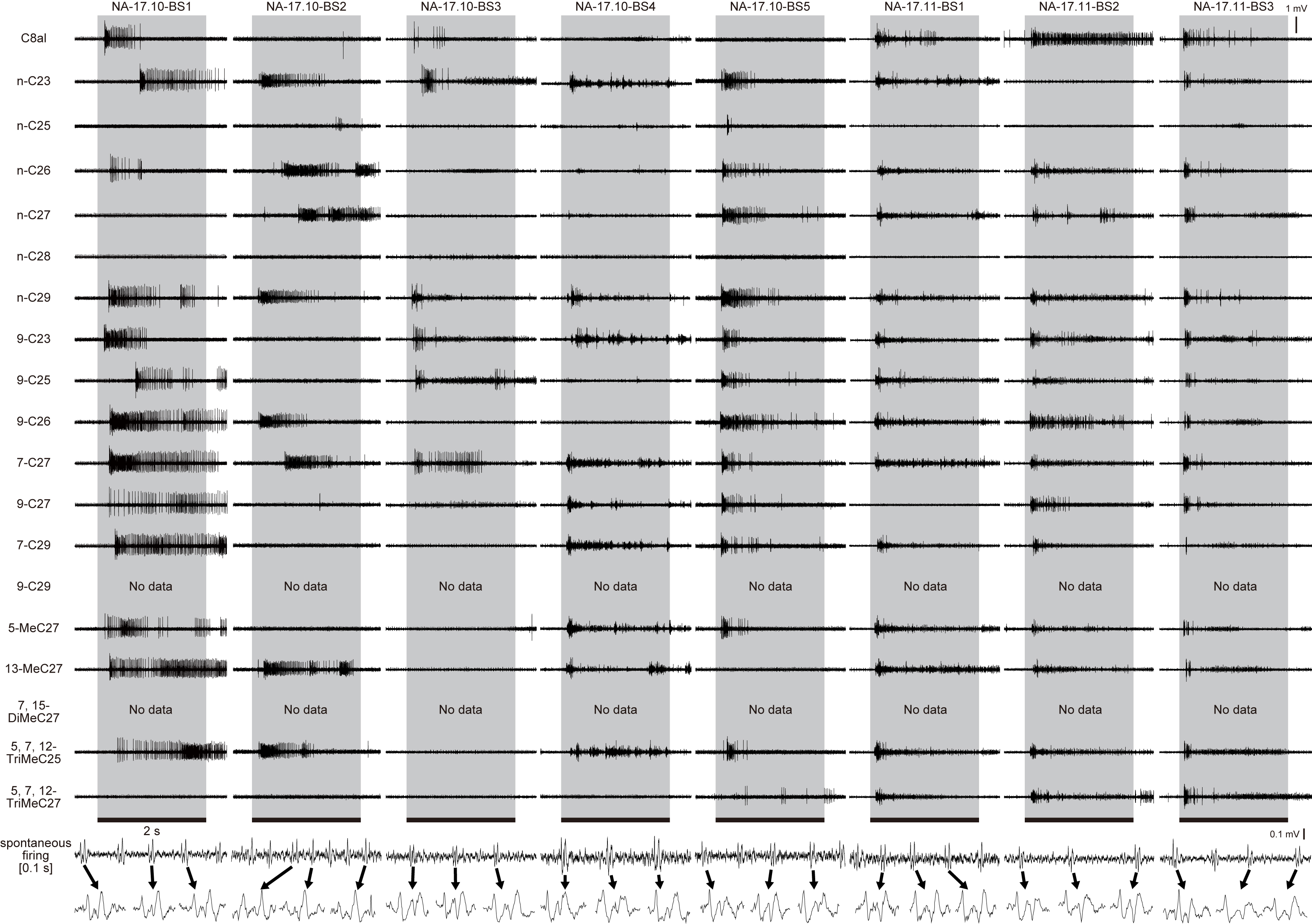

Supplement: Supplementary Figures 3–5 — Cuticular hydrocarbon (CHC) responses of 23 basiconic sensilla analyzed in this study. Each basiconic sensillum exhibited “all-or-nothing” responses to tested CHCs. In each sensillum, each of large spontaneous spikes are temporally synchronized with several small spikes. Recorded basiconic sensilla are termed by “colony name (NA, NB, or NC)–sampling date (year. month)–sensillum number (BS1-6).” The 2-s CHC stimuli are indicated by gray boxes and horizontal bars under the electrophysiological traces. [file Image_3.JPEG]
